# Rapid, Long-Distance Dispersal by Pumice Rafting

**DOI:** 10.1371/journal.pone.0040583

**Published:** 2012-07-18

**Authors:** Scott E. Bryan, Alex G. Cook, Jason P. Evans, Kerry Hebden, Lucy Hurrey, Peter Colls, John S. Jell, Dion Weatherley, Jennifer Firn

**Affiliations:** 1 School of Geography, Geology and Environment, Kingston University, Kingston Upon Thames, Surrey, United Kingdom; 2 Earth, Environmental and Biological Sciences, Queensland University of Technology, Brisbane, Queensland, Australia; 3 Queensland Museum, South Brisbane, Queensland, Australia; 4 Department of Geology and Geophysics, Yale University, New Haven, Connecticut, United States of America; 5 Climate Change Research Centre, University of New South Wales, Sydney, Australia; 6 School of Agriculture and Food Sciences, The University of Queensland, St Lucia, Queensland, Australia; 7 School of Earth Sciences, The University of Queensland, St Lucia, Queensland, Australia; 8 Petrographic International, Clontarf, Queensland, Australia; 9 WH Bryan Mining and Geology Research Centre, The University of Queensland, St Lucia, Queensland, Australia; University of Leeds, United Kingdom

## Abstract

Pumice is an extremely effective rafting agent that can dramatically increase the dispersal range of a variety of marine organisms and connect isolated shallow marine and coastal ecosystems. Here we report on a significant recent pumice rafting and long-distance dispersal event that occurred across the southwest Pacific following the 2006 explosive eruption of Home Reef Volcano in Tonga. We have constrained the trajectory, and rate, biomass and biodiversity of transfer, discovering more than 80 species and a substantial biomass underwent a >5000 km journey in 7–8 months. Differing microenvironmental conditions on the pumice, caused by relative stability of clasts at the sea surface, promoted diversity in biotic recruitment. Our findings emphasise pumice rafting as an important process facilitating the distribution of marine life, which have implications for colonisation processes and success, the management of sensitive marine environments, and invasive pest species.

## Introduction

**Figure 1:**
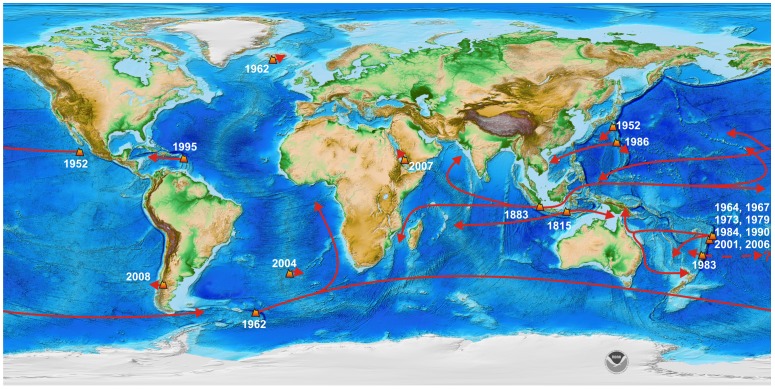
Significant pumice rafting events over the last 200 years. Volcanic eruption locations, eruption dates and general trajectory paths of pumice rafts are shown illustrating the global scale and frequency of such events. To maintain figure clarity, only pumice raft-producing eruptions for the last 50 years from the Tonga-Kermadec arc, (southwest Pacific) are listed. Data sources are given in Supporting Information (Appendix S1) to this paper. Base map is from Amante and Eakins [14].

Pumice rafting is an important, but poorly understood and little known natural phenomenon that reflects a dynamic interplay between volcanism, the atmosphere and oceans, and marine biology. Such geological rafts have been suggested as a long-distance dispersal mechanism that can overcome physiological limitations on dispersal ranges for many marine species; they provide intermittent contact between shallow marine and coastal ecosystems that otherwise remain isolated by vast stretches of deep ocean [1]–[6]. However, long-distance rafting or dispersal events have rarely been observed or quantified such that an understanding of their mechanisms, trajectories, influencing factors, and magnitude is lacking [7]. It is assumed that many rafting substrata either have short lifespans [5] due to biological and/or physical destruction, or are produced by episodic, low frequency events (e.g., volcanic eruptions) that minimises propagule pressure [8] and establishment success; the consequence being that rafting is not widely considered in studies focussing on marine invasive biology or population connectivity, which is thought to be principally achieved and maintained by pelagic larval dispersal (e.g., [9]–[11]). However, a survey of recent volcanic eruptions reveals that pumice rafts have occurred in all the major oceans over the last 200 years (Fig. 1), and throughout the Holocene, but are particularly high frequency events in the Pacific Ocean. In this study we present the first-ever, systematic documentation of the biological cargo of a pumice raft using pumice material produced by the 2006 explosive eruption of Home Reef Volcano in Tonga [12], 13, and then collected from ocean waters and islands in Tonga and cast ashore in eastern Australia (Table 1), up to 900 days after the eruption.

**Table 1:** Pumice strand sample sites, Eastern Australia.

Sample Site	Latitude	Longitude	Type of materialcollected	Sampling Date	Number of clastsexamined
Whitsunday Island	20°S 17.669′	149°E 03.249′	Representative	29/4/2007	−
Marion Reef	19°S 05.744′	152°E 23.449′	Representative	30/4/2007	50
Lamberts Beach	21°S 04.472′	149°E 13.701′	1 m^2^ quadrat; representative	1/5/2007	806
Mackay Harbour	21°S 07.434′	149°E 13.277′	Representative	1/5/2007	−
Salonika Beach	21°S 18.300′	149°E 17.605′	Representative	1/5/2007	−
Lady Musgrave Island	23°S 54.461′	152°E 23.669′	1 m^2^ quadrat; representative	3/5/2007	1545
Agnes Waters	24°S 12.463′	151°E 54.364′	Representative	3/5/2007	120
South Stradbroke Island	27°S 49.678′	153°E 25.968′	Representative	1/6/2007	200
Broadbeach	28°S 07.620′	153°E 26.135′	1 m^2^ quadrats; representative	5/5/2007; 27/12/2007; 2/1/2008	390
Duranbah	28°S 10.005′	153°E 33.105′	1 m^2^ quadrat; representative	5/5/2007	216
Byron Bay	28°S 38.334′	153°E 37.636′	Representative	5/5/2007	
Tallow Beach, Byron Bay	28°S 38.760′	153°E 37.921′	1 m^2^ quadrat	5/5/2007	710
Shelley Beach, Ballina	28°S 51.598′	153°E 35.795′	1 m^2^ quadrat	5/5/2007	806

### The 2006 Eruption of Home Reef and Pumice Rafts

After 22 years of dormancy, a category 2–3 (Volcanic Explosive Index) dacitic eruption at Home Reef from 7–16 August 2006 produced a new but temporary volcanic island (pumice cone) and a large floating mass of pumice initially extending over >440 km^2^ [12], [13]. Like many of the historical Tongan eruptions, there were few direct observations of the main phase of the eruption and no obvious precursory signals to the new unrest at Home Reef. Few details were available on the form or structure of this submarine volcano prior to the 2006 eruption, although the volcano summit was considered to be ∼10 m below the surface given that the 1984 eruption had also produced a temporary pumice island [15]. Restricted observation of the eruption from the nearby Vava’u islands (∼75 km to the ENE) suggested a subplinian eruption column developed at the onset of the eruption, rising to heights of 7–15 km, and was sustained for at least a few hours. The main eruption appears to have been driven principally by magmatic explosivity, with hot pumice and ash largely excluded from the shallow water column by the erupting jet. Airborne cooling of the pumice in the eruption column was therefore important to cool pumice to form the floating pumice raft [4] as experimental studies have shown hot pumice rapidly ingests water, becomes negatively buoyant and sinks [16]. The growth of an emergent pumice cone at the vent, estimated to be up to 75 m high [12] is consistent with observations from recent subaerial explosive eruptions for significant vent overthickening of pyroclastic deposits (e.g., ref. [17], [18]). Rapid removal of this pumice cone and island by wave action in the ensuing 8 months demonstrated that deposition around the vent was entirely of unconsolidated pyroclastic material. Episodic surtseyan explosive jets persisted for several days after the August 7 eruption [12], reflecting some explosive interaction with seawater. Significant SO_2_ emissions (∼25 kilotons) were measured during the subplinian phase [12], and ongoing observation of Home Reef by us confirmed continued SO_2_ degassing and hydrothermal venting until at least December 2008, 28 months after the eruption.

The large pumice raft produced in the eruption on August 7 2006, moved northeast towards the Vava’u Islands of northern Tonga and subsequently headed northwest and westwards reaching Fiji by mid-September 2006 [12], [13]. By this time, the pumice raft had become dispersed forming extensive stringers or windrows, tens to hundreds of kilometres long, over a much increased area of ocean (∼1600 km^2^ [13]). Around the same time, the first reporting of organisms (goose barnacles, *Lepas sp.*) attached to the pumice was made [12]. Pumice strandings on islands in Vanuatu [19] and then New Caledonia from November 2006 to January 2007 recorded the continued passage and dispersal of the pumice westwards until it reached eastern Australian waters and the Great Barrier Reef World Heritage Area by March 2007, approximately 200 days after the eruption. Repeated pumice strandings occurred along the eastern Australian coastline from March 2007 to April 2008 (20 months after the eruption), testifying to the duration that pumice can remain afloat in ocean waters.

**Figure 2:**
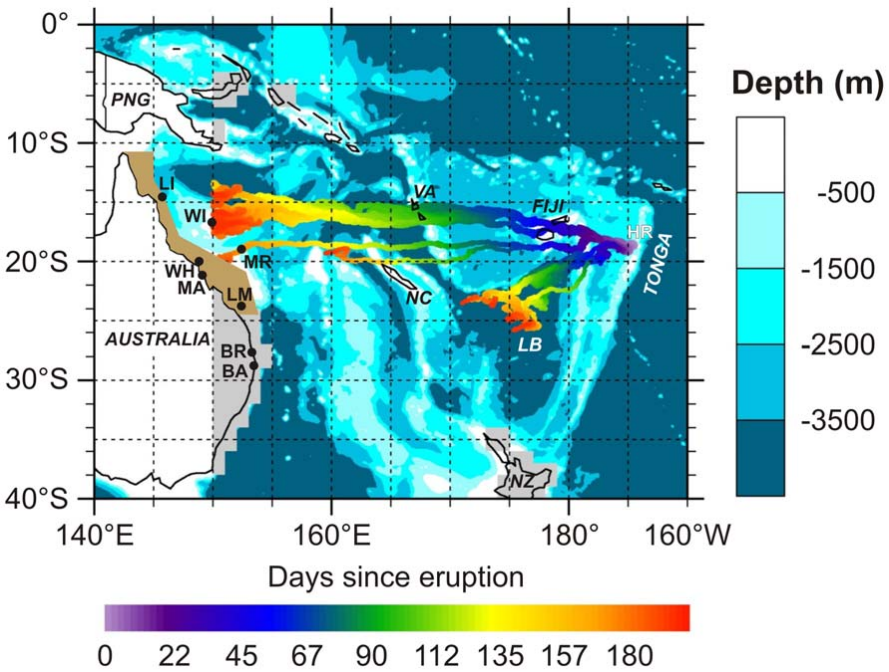
Trajectory map of the 2006–2007 pumice rafts, based on the integrated surface velocity field. Pumice strandings following the Home Reef (HR) eruption were reported at the following locations: Fiji (∼33 days); Vanuatu (VA, 88 days); New Caledonia (NC, ∼115 days); Willis Island (WI, ∼180 days); Lizard Island (LI, ∼200 days); Mackay (MA, ∼250 days); Broadbeach (BR, ∼250 days). Other abbreviations: LB, Lau Basin; NZ, New Zealand; PNG, Papua New Guinea; MR, Marion Reef; LM, Lady Musgrave Reef; WH, Whitsunday Island; BA, Ballina. Brown shaded region along northeastern Australia is the Great Barrier Reef World Heritage Area. Grey areas without bathymetric information represent continental shelves of <1000 m depth, where geostrophic ocean currents were not calculated. An animated version of the pumice raft trajectory is provided in Supporting Information (Figure S1) to this paper.

**Table 2:** Quantitative data for epibionts transported by the 2006–2007 pumice rafts.

Epibiont	Average number of individuals/100 clasts ± SD	Range of number of individuals/clast
Gastropods (mainly *Recluzia* sp.)	34±132	0–20
Goose barnacles (*L.* anserifera)	79±475	0–234
Fouling cheilostomes (mainly *Jellyella* sp.)	256±308	0–15
Serpulids	19±151	0–63
Bivalves (mainly *Pteria, Pinctada* sp.)	1±15	0–6
Bivalves oysters (*Crassostrea* sp.)	0.5±9	0–5
Encrusting forams	9±132	0–65
Corals (mainly *Pocillopora* sp.)	1±12	0–6
Anemones (incl. *Calliactus* sp.)	0.3±6	0–2
Egg casings (incl. *Halobates* sp.)	4±29	0–6
Isopods/amphipods (mainly *Ianiropis* sp.)	1.5±18	0–6
Sponges (Porifera)	0.2±4	0–1

The number of individuals is based on descriptions of 4984 clasts collected from locations listed in Table 1.

**Table 3:** Quantitative data for colonial epibionts transported by the 2006–2007 pumice rafts.

Colonial Epibiont	% Coverage of pumice clast surface ± SD	Range in % coverage/clast
Cyanobacteria (mainly *Rivularia* spp.)	28.4±27	0–100
Macroalgae (includes *Caulerpa, Jania, Polysiphonia, Colpomenia, Calithamnion, Sargassum* sp.)	1.1±5	0–80
Calcareous algae	1±3	0–80
Cheilostome Bryozoa (mainly *Jellyella* sp.)	7.5±15	0–95

Areal coverage is based on descriptions of 4984 clasts collected from locations listed in Table 1.

**Table 4:** Temporal variation in total epibiont coverage.

Epibiont Coverage	% Coverage of pumice clast surface ± SD	Range in % coverage/clast
April-May 2007	33±30	0–100
December 2007-January 2008	79±23	3–100

Samples collected from eastern Australia following the April stranding event and then at the end of December 2007 and beginning of January 2008 are compared.

## Results

### The Rafting Substratum

Pumice is an extremely effective rafting agent that can dramatically increase the dispersal range of a variety of marine organisms [1], [2], [4]. The physical properties of pumice result in it being resistant to biological consumption and physical weathering. Our observations of active rafts, collected pumice material and simple flotation experiments (see also ref. [20]) using cold pumice from the 2006 Home Reef eruption indicate positive buoyancy of pumice is aided and maintained by: 1) primary vesiculation heterogeneities within individual clasts that reduce clast permeabilities; 2) flotation with freeboard that reduces the effective permeability and rate of waterlogging; 3) a temporal reduction in clast permeability by encrusting organisms such as Bryozoa and; 4) algal and cyanobacterial respiration aerating pore/vesicle spaces. The highly vesicular and porous nature of pumice ensures that it offers a high surface area to size ratio and space for attachment [4], [21]. Vesicles and surface depressions offer protection from predation for obligate rafting organisms and for facultative species during initial growth. Pumice has global sources (volcanoes) and given its longevity as a floating object (months to years [4], [20], [22]), it can be globally distributed, unrestricted by ocean temperatures or climatic variations or ocean basins (Fig. 1). However, its potentially greatest asset as a rafting vehicle may be the sheer volume and mass of pumice that is introduced into oceans following volcanic eruptions. We estimate the number of pumice clasts produced by the fragmentation of ∼0.16 km^3^ magma in the Home Reef eruption to be >2.5×10^12^ (see Materials and Methods). Importantly, each clast is a potential raft opportunity for an organism, emphasising the sheer abundance of rafting vehicles available immediately following a volcanic eruption.

**Table 5:** Summary of epibiont taxa, their designated feeding guild and their averaged frequency of occurrence.

Epibiont Order or Clade* (number oftaxonomic units)	Feeding Guild	Average Frequency of Occurrence
Cheilostomata (5)	Suspension/filter feeder	42%
Pedunculata (1)	Suspension/filter feeder	22%
Hypsogastropoda* (2)	Predator/scavenger; grazer/borer;	13%
Littorinimorpha* (2)	Grazer/borer	
Ptenoglossa (2)	Predator/scavenger	
Sorbeoconcha* (1)	Grazer/borer	
Leptomedusae (3)	Suspension/filter feeder	5%
Coronatae (2)	Suspension/filter feeder	
Canalipalpata (2)	Suspension/filter feeder	5%
Rotaliida (2)	Suspension/filter feeder	2%
Polythalamea (2)	Suspension/filter feeder	
Amphipoda (1)	Predator/scavenger	1%
Isopoda (1)	Predator/scavenger	
Actiniaria (2)	Suspension/filter feeder	<1%
Amphinomida (3)	Predator/scavenger	<1%
Decapoda (3)	Predator/scavenger	<1%
Dictyoceratida (3)	Suspension/filter feeder	<1%
Egg casings (3)		<1%
Nudibranchia* (2)	Predator/scavenger	<1%
Ostreoida (3)	Suspension/filter feeder	<1%
Pterioida (6)	Suspension/filter feeder	<1%
Scleractinia (>2)	Suspension/filter feeder	<1%
Sessilia (1)	Suspension/filter feeder	<1%
Photosynthetic Groups		
Cyanobacteria (5)		89%
Calcareous algae (4)		35%
Macroalgae (17)		19%

Total number of taxonomic units listed in parentheses is 80.

**Figure 3:**
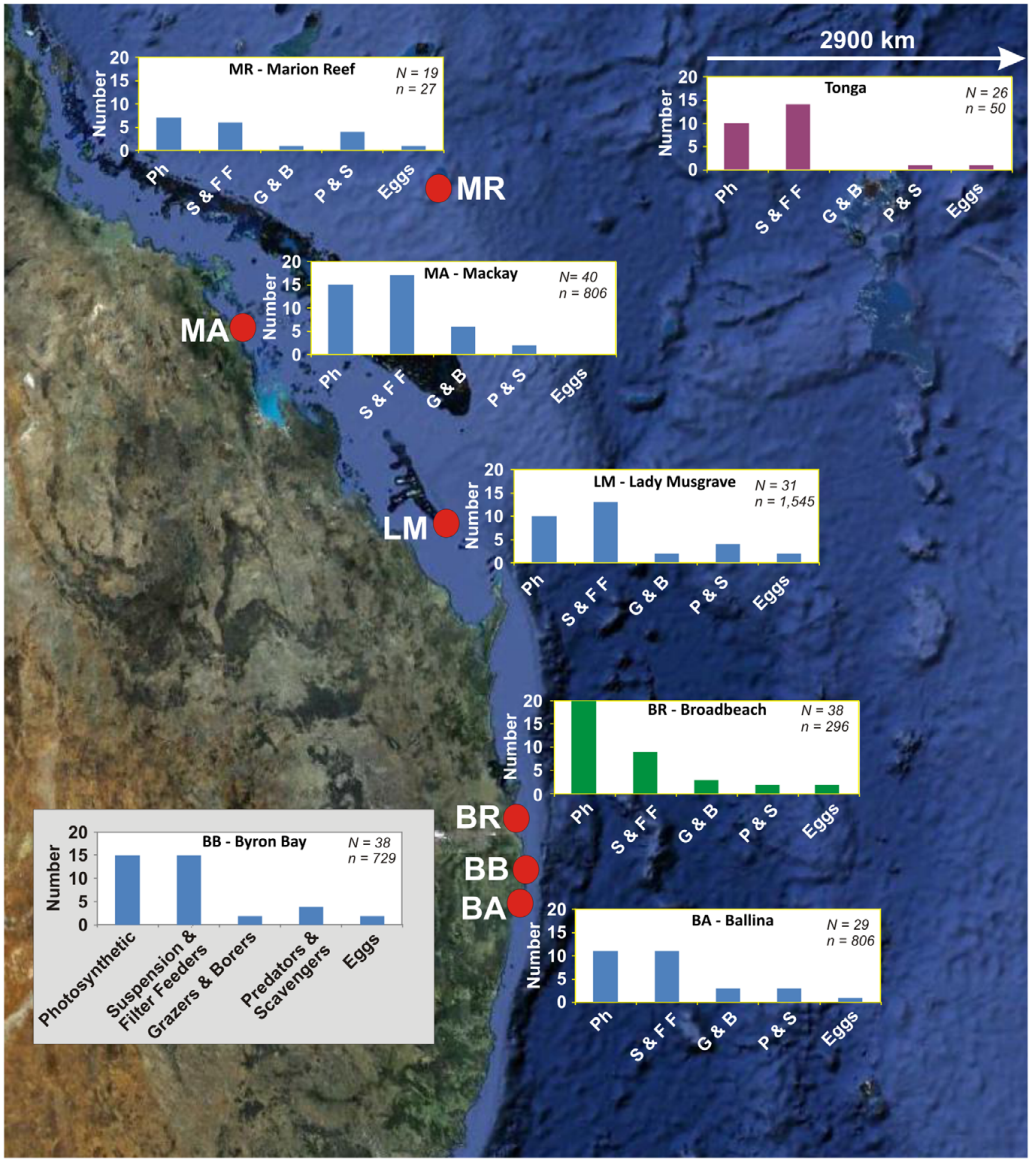
Proportions of rafted epibionts along the trajectory. Number refers to number of taxonomic units identified at each sample site. Marine invertebrates are grouped in terms of feeding behaviours. Suspension and filter feeders (e.g., cheilostome Bryozoa, goose barnacles, hydroids/scyphozoans, serpulids, corals, molluscs, and oysters) show significant early recruitment (Tonga) with epibiont diversity generally maintained along the raft trajectory. The numbers of plants (cyanobacteria, macroalgae and calcareous algae) increased with time and along the trajectory, particularly once pumice had arrived into eastern Australian waters. Overall, epibiont diversity increased with time. Bar graphs are colour-coded with respect to observation/collection timing: purple, February 2007; blue, April-May 2007 and; green, December 2007. *N* is total number of species/taxonomic units observed, and *n* is number of pumice clasts described from each location. Abbreviations: Ph, photosynthetic; S & FF, suspension & filter feeders; G & B, grazers & borers; P & S, predators and scavengers. Locations: MR, Marion Reef; MA, Mackay; LM, Lady Musgrave; BR, Broadbeach; BB, Byron Bay; BA, Ballina. Tonga sample site occurs ∼2900 km to the east. Base map from Google Earth.

### Rate of Transport of Biological Community Provided by Pumice Rafts

For rafting to be successful, distances travelled and frequency of dispersal events are of principal importance, whereas rate, transport direction and duration of rafts can significantly impact on rafted taxa abundance and diversity [23]. In the southwest Pacific Ocean, floating objects are driven westwards by the prevailing winds and equatorial ocean currents, resulting in their accumulation in eastern Australian waters. Knowledge of the trajectory taken by pumice sourced from volcanoes in the Tonga-Kermadec region is important in order to constrain the location and timing of island and reef encounters from which shallow marine organisms can be recruited for long-distance transport. The timing of reef encounters can be particularly important to enable and maximise recruitment of larvae during seasonal or monthly spawning events (e.g., corals).

Drift trajectories of Tonga-derived pumice have been mapped using observations and sightings of stranded pumice [12], [13], [19], and computed using numerical models of southwest Pacific wind fields and ocean currents (Fig. 2) as described in Materials & Methods. Pumice raft trajectory is a combination of surface currents, wave motions and direct wind drag. The 2006–2007 pumice raft trajectory was not disturbed by cyclonic activity, which was in contrast to that of the pumice rafts originating from the 2001 eruption of the nearby unnamed submarine volcano 0403–091 [4]. However, relatively strong and persistent trade winds resulted in strong dismemberment of the pumice raft, particularly early on along the trajectory (Fig. 2). The main pumice trajectory passed the Fiji islands and then between Vanuatu and New Caledonia, while a secondary mass separated approximately a month after the eruption and dispersed to the southwest and into the Lau Basin. The main pumice rafts arrived in eastern Australian waters ∼7 months after eruption, ultimately travelling >5000 km to reach Australia and potentially, Papua New Guinea [19], [24]. Mean speed (current + winds) of the pumice rafts westwards was ∼0.23 m s^−1^ (∼20 km day^−1^; cf. ref. [25]) - twice as fast as the mean current velocity experienced by the rafts on their journey (∼0.11 m s^−1^). This drift rate, by utilising surface currents, is significant when compared to the potential drift rates and distances of pelagic larvae [9], [26], [27]. Consequently, recruitment onto pumice counteracts strategies promoting local retention and replenishment of source populations [11], [28], or reductions in larval exchange due to mortality and diffusion [29].

**Figure 4:**
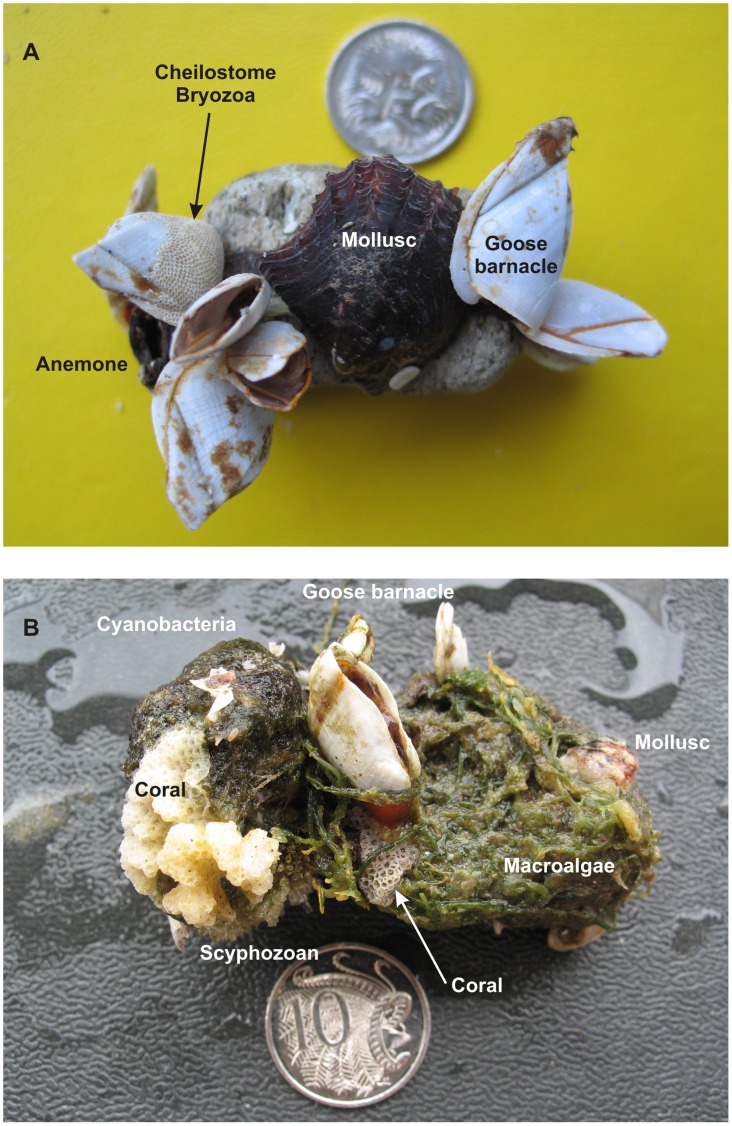
Epibiont colonisation on Home Reef pumice. (**A**) Pumice clast collected from Marion Reef on April 30, 2007 with a mature epibiont fauna attached. Based on compiled growth rates [23], the largest goose barnacles (*Lepas anserifera*; 23 mm length) have been attached to the pumice for a minimum of 60 days, while the size of the mollusc indicates up to 200 days of growth [33]. Note the rounded and abraded form of the pumice clast on to which the epibionts have attached. Coin is 2 cm diameter. (**B**) Heavily fouled pumice collected from a secondary stranding at Broadbeach, southeastern Queensland on December 27, 2007 (807 days after the eruption). Two pumice clasts are bound together by cyanobacteria (principally *Rivularia* sp.) and macroalgae (*Caulerpa* sp.) with two corals (*Pocillopora* sp.), a colonial scyphozoan (Order Coronatae), goose barnacles (*Lepas anserifera*) and mollusc (*Pinctada* sp.) also attached. Coin is 2.4 cm diameter.

**Figure 5:**
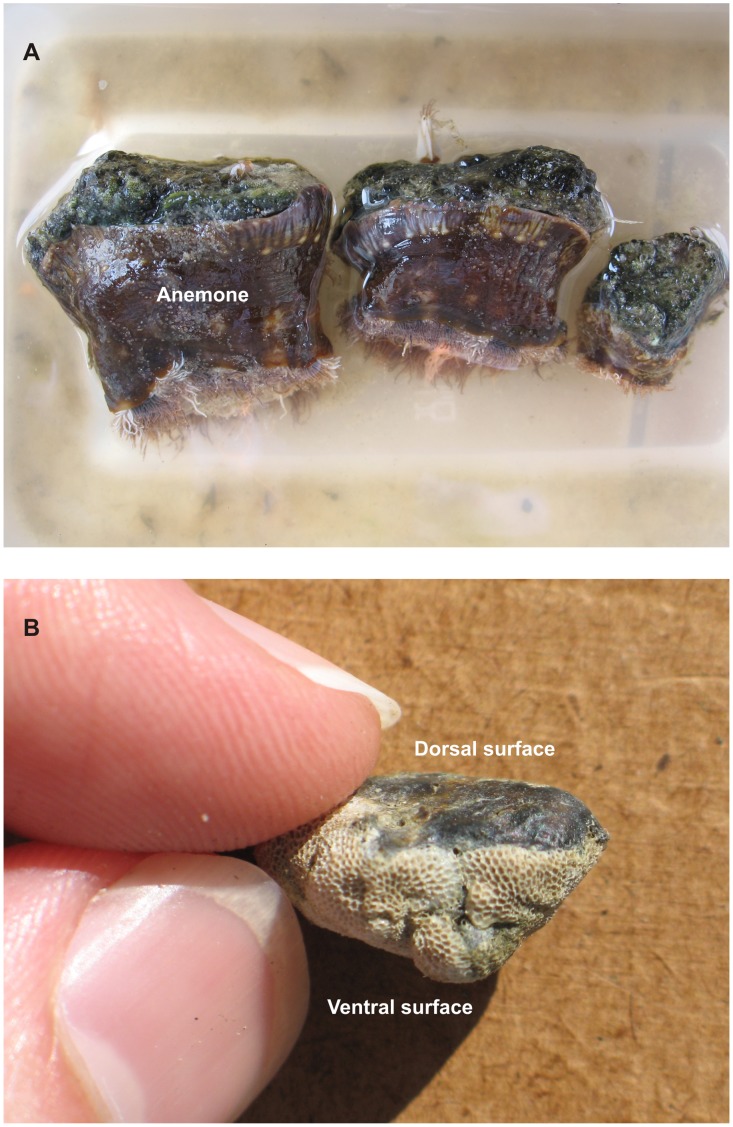
Epibiont distribution on Home Reef pumice. (**A**) Three pumice clasts collected from Broadbeach on December 27, 2007 with well-developed biological keels of the anemone *Calliactus* sp. with cheilostome Bryozoa (*Jellyella* sp.) along the waterline and *Rivularia* spp. occupying all of the dorsal surface with occasional goose barnacles (*Lepas anserifera*); pumice clast at left is 5 cm long. (**B**) Typical observed polarity in epibiont distribution on pumice with dorsal surfaces almost exclusively occupied by cyanobacteria (*Rivularia* sp.), and here, the ventral surface entirely covered by cheilostome Bryozoa (*Jellyella* sp.) colonies. Clast is 1.7 cm long, collected from Lamberts Beach, Mackay.

**Table 6:** ANOSIM results.

Collection/Arrival Time comparison	Species presence & absence	Species abundance
Global	0.23 (0.001)	0.03 (0.01)
Late, Early	0.51 (0.001)	0.20 (0.001)
Late, Middle	0.28 (0.001)	0.009 (0.30)
Early, Middle	0.153 (0.001)	0.09 (0.001)

Global R statistics and P-values in brackets, with results of pairwise tests of significance depending on collection/arrival time for response variables of species presence and absence, and species abundance listed separately.

**Figure 6:**
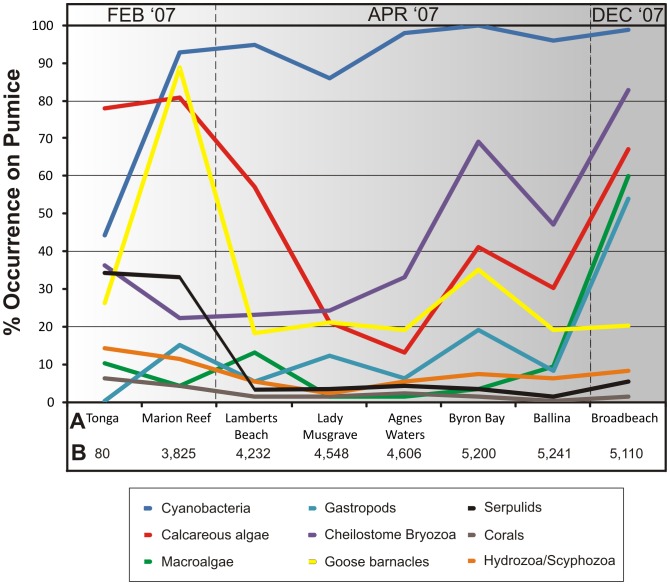
Biotic changes of the Home Reef pumice rafts during 2006 and 2007. Frequency of occurrence (expressed as %) of biota on pumice clasts is shown relative to pumice raft arrival time, sample location (A) and approximate distance along the trajectory in kilometres (B), which correlates with floating time. Three general epibiont trends are observed with time: 1) rapid colonisation of all available pumice resulting in ubiquity (cyanobacteria) – a few sample sites showed slightly reduced occurrences of cyanobacteria on pumice (e.g., Lady Musgrave), but this resulted from clast abrasion across reefs, followed by some post-stranding desiccation and spalling; 2) a progressive increase in occurrence with time (e.g., cheilostome Bryozoa, gastropods and macroalgae) and; 3) stalled colonisation where some species were successfully recruited early on to pumice but underwent no further colonisation expansion due to insufficient time to reach sexual maturity (corals), or the epibionts continued to colonise the same clast (serpulids, hydrozoans/scyphozoans), increasing the numbers of conspecifics per clast; these taxa also had relatively low initial recruitment numbers.

### Abundance of Organisms Transported

Successful dispersal not only depends on transport direction and velocity of floating items, but also on their total abundance in a particular region [6]. Furthermore, high propagule pressure (the number of individuals arriving in any one event and the number of discrete arrival/release events) facilitates invasions and establishment success [8], [30]. An important outcome of the trajectory modelling (Fig. 2) is that up to two-thirds of the initial pumice raft material is indicated to have reached eastern Australian waters. However, modelling cannot take into account losses along the trajectory through waterlogging, biotic overloading or island strandings, or increases in abundance through clast breakage. Losses through waterlogging [16] or overloading by fouling organisms [22] were minimal given the average clast size of pumice reaching eastern Australia was 1–2 cm (1.4±1 cm maximum length × 0.8±0.6 cm minimum length, mean ± SD) and the relatively short duration of pumice flotation (∼7 months). Island strandings could have contributed significantly to reducing the flux of pumice to eastern Australia. We therefore make the very conservative estimate that one-third of the pumice raft material reached eastern Australian waters, which equates to ∼8.3×10^11^ clasts. Most significantly, each one of these pumice clasts represents a rafting opportunity for organism(s).

Positive relationships exist between raft size and number of travellers [23]. The proportion of clasts with marine invertebrates was high (>50%), and our studies indicate that any limitations enforced by clast size (maximum observed size was 24 cm diameter) were overcome by the sheer number of pumice clasts produced during the eruption. We also found the total rafted biomass was substantial and increased with time, concomitant with a biodiversity increase. Numbers of individuals and percentage coverage (Tables 2,3,4) of pumice by organisms give some insight into the amount of biota transported. For some taxa, the average number of individuals per 100 clasts demonstrates the substantial mass of faunal transfer, despite considerable variance in the numbers of organisms between clasts observed at each sample site (Table 2).

Goose barnacles (*Lepas anserifera*) were prolific in the early infestation of pumice, with some pumice lapilli collected from ‘live’ rafts in Tonga carrying >220 individuals. These numbers (Tables 2,3), given the estimated number of individual pumice clasts produced by the eruption (>2.5×10^12^) and surviving transport to eastern Australia (∼8×10^11^), translate into the long-distance rafting of >10 billion individuals or colonies for some taxa. In some cases, the numbers of individuals rafted will have increased along the raft trajectory because several species will have reached sexual maturity during the rafting event (e.g., goose barnacles). These data thus indicate a large biomass is ferried during pumice rafting events, especially in tropical waters resulting in high propagule pressure for many taxa. The high population numbers indicated here (Tables 2,3) have fundamental implications for increasing the genetic diversity of the rafted population and founder populations they may contribute to, as the number of conspecifics that will arrive simultaneously will greatly enhance the establishment and persistence of new populations [6], [8], [23], [30], [31].

### Diversity of Biological Cargo

Previous observations have recorded a relatively depauperate community on pumice (e.g., ref. [3]). These results are based, however, on studies of pumice that have resided for long periods on beaches where only organisms with calcareous skeletons remained or where observations of recruitment have been made in temperate ocean waters [22]. In addition, because pumice offers no nutritional value, a low biodiversity may result and the recruitment of species capable of exploiting allochthonous food sources is promoted [23]. Our data (Fig. 3), based on examining living pumice rafts in tropical waters and newly stranded material, suggest that on pumice, assemblages quickly mature and become relatively bioresource-rich enabling a diverse community to develop (>80 species, Table 5), more than previously recognised. The rafted community exhibits a variety of feeding strategies: photosynthetic, filter feeding, grazing and scavenging to predation, but with photosynthesising organisms and filter feeders most dominant (Fig. 3, Table 5). We note that the abundance of motile organisms was strongly biased by sampling timing: samples collected from live rafts or newly stranded deposits had higher abundances of self-propelled organisms (nudibranchs, isopods, amphipods, polychaete worms and crabs), which disembarked from the pumice after stranding.

Unlike macroalgae rafts that may carry with them pre-detachment original inhabitants, the pumice rafts had an initial period of sterility lasting a few weeks, before a drift community became quickly established on the pumice. This pumice-based community then continued to grow and diversify over the life of the pumice rafting event. We are able to discriminate rafted biota into “early”, “middle” and “late” successional stages based on sampling along the raft trajectory, and comparison to epibiont growth sizes [23], [32]–[35]. These assemblages also have spatial significance given the trajectory from Tonga to eastern Australia, and the prolonged residence time for over a year in eastern Australian waters. Comparisons between February-April 2007 and December 2007 collected material have been particularly instructive in revealing how the epibiont assemblage matured. Goose barnacles, cyanobacteria, cheilostome Bryozoa, calcareous algae, serpulids, and to a lesser extent, macroalgae (*Hypoglossum* sp., *Polysiphonia* sp.), nudibranchs and hydroids/scyphozoa formed a proximal or early colonising assemblage (attachment within 2 months and locally around Tonga). Continued biotic recruitment of corals, bivalves, serpulids, anemones (Figs 4,5), macroalgae (particularly *Ceramium* sp., *Sargassum* sp.), cyanobacterial colonies of Order Oscillatoriales, gastropods (dominantly *Recluzia* sp.) and oysters (*Crassostrea* spp), during the pumice raft voyage from Tonga to Australia (attachment between 2–7 months), added to the proximally and early recruited assemblage, to form the middle colonising assemblage. The late assemblage continued this diversification trend, recruiting organisms from tropical and subtropical waters in eastern Australia from March to December 2007 with new recruits including macroalgae (particularly several species of *Caulerpa*, Fig. 4B), scyphozoans, sponges, acorn barnacles, arthropods, and bristle worms; numerous *Halobates* eggs were also found attached to pumice.

To support our observations of successional stages in the rafted taxa, we undertook an analysis of similarity (Table 6) focussing on the presence and absence of taxa (richness) and their relative abundances to evaluate how raft communities were changing depending on the arrival and collection time of the pumice rafts. We found early (<7 months after eruption), middle (∼9 months after eruption) and late (16 months after eruption) pumice rafts were significantly different in terms of species richness. Species abundance values also differed between rafts depending on arrival time but differences were not as strong as with species richness. Early and late pumice raft strandings had the strongest differences in term of species richness, and species abundance (Table 6). Late and middle arriving rafts were found to differ significantly in terms of species richness, but not species abundance. Early and middle raft biota were the most similar in terms of species richness, although still significantly different in species abundance.

Biotic succession involving the disappearance of early attached biota is not an obvious feature of the 2006–2007 rafting event. All previously attached biota continued to grow and survive along the pumice trajectory (Fig. 6), with the degree of coverage of pumice increasing with time attaining >75% coverage of clasts by December 2007 (16 months after the eruption), as well as much reduced variation in epibiont coverage (Table 4). Cheilostome Bryozoa, gastropod and macroalgal occurrences on pumice are particularly noteworthy for significant increases from April 2007 to December 2007 (Fig. 6). Instead, and more importantly, polarity in epibiont distribution on pumice clasts (Fig. 5) developed during rafting as a result of the stability of pumice clasts at the sea surface (see ref. [22]). Dorsal sides of pumice clasts were almost exclusively occupied by cyanobacteria (dominated by *Rivularia spp*), calcareous algae, and occasionally macroalgae. Epibiont paucity on the dorsal sides of pumice clasts largely reflects the exposed surface environment due to the pumice floating with some freeboard, resulting in persistent solar radiation and to a lesser extent, air exposure. Solar radiation and desiccation have been reported to negatively affect colonization of littoral benthic communities [36], [37]. In contrast, ventral sides, which are more shaded and continually submerged, developed the greatest biodiversity and prominent biological keels of predominantly cheilostome Bryozoa, goose barnacles, corals, bivalves, anemones (Fig. 5), macroalgae, gastropods, serpulids and hydroids/scyphozoa. Over time, the ventral epibiont assemblage, by forming biological keels, reinforced pumice clast stability and thus the differing microenvironmental conditions on opposite sides of the pumice clasts. Microenvironmental conditions therefore played an important role in limiting the ability of one species to monopolise each clast. Nevertheless, even the smallest clast population (<1 cm diameter) was duopolized by cyanobacteria and cheilostome Bryozoa, often exclusively occupying the dorsal and ventral clast surfaces, respectively (Fig. 5B).

## Discussion

Attention to transport and connectivity issues of marine communities has increased dramatically in the past decade, driven by concerns over the spread of invasive species, marine reserve design for improved conservation, fisheries resources, and climate-change effects [38]. We conclude that pumice rafting events, even following small-volume eruptions like the 2006 eruption of Home Reef, are very important recruitment and dispersal events, based on: 1) their encounters with islands, reefs and other biologically diverse shallow marine coastal environments, further enhanced by coinciding with episodic spawning events; 2) the sheer abundance of pumice produced by explosive eruptions and which can survive long-distance transport and remain afloat for months to years; 3) raft velocities (approximately twice as fast as the mean ocean current velocity, due to the utilisation of surface currents) and; 4) the substantial biomass and biotic diversity observed here to have been rafted thousands of kilometres. This has several important implications. Pumice rafting fundamentally changes the dispersal range and limitations for many marine taxa, particularly those with short pelagic larval stages or where controls exist on larval supply or where larval behaviour may influence dispersal [28], [38], . Pumice rafting of organisms, whilst temporally random over short time frames, is a consistent and effective measure of transporting organisms over large distances and across deep ocean basins. Given the volume of pumice mobilised, pumice rafting is a mass transit process unrivalled by any other rafting substrata. Volcanic eruptions appear to have an elevated frequency in the SW Pacific [40], and historically, pumice rafts have occurred approximately once every ten years promoting enhanced population connectivity for scores of shallow marine species in this region. At the global scale (Fig. 1), there is little basis to consider that pumice rafting is a low frequency event that would reduce the likelihood of successful transport on this substratum (cf. ref. [22]). For the SW Pacific, pumice rafting is not only a recent phenomenon, and the modern Tonga-Kermadec Arc has been active since at least 2 Ma [41]. The success of these dispersal events in the SW Pacific is further enhanced by the raft trajectory, along which exist many coral reef habitats for recruitment and representing suitable and similar habitats for colonisation [23]. Our results are consistent with previous studies in the region that suggest massive transport of genetic material occurs from east to west and that for corals is from an area of low to high diversity [2]. This is because the geographic distribution of tropical shallow marine species is being strongly controlled by ocean/surface current patterns [42], and enhanced by geologic events. Pumice rafts, as they enter tropical eastern Australian waters, and then move both north and south following the East Australian Current, provide lines of internal communication for the Great Barrier Reef World Heritage Area. Given that recruitment largely occurs from oceanic reefs and the main reef-building organisms of corals, Bryozoans and calcareous algae are found in abundance on the pumice (Tables 3,5), pumice rafts may offer a natural process for restocking reefs damaged from either natural or anthropogenic causes. Finally, pumice rafts present biosecurity concerns as they represent a potential vector for invasive species. Even if infestation rates of a pumice raft by a marine pest are extremely low (e.g., one of the lowest measured occurrence rates was for sponges at 0.002% and some sponges can be a marine pest), this can still translate to the long-distance transport and invasion by millions to billions of individuals, for which current mitigation measures are not designed for.

## Materials and Methods

### Sampling

Pumice raft material produced by the 2006 Home Reef eruption and examined in this study was collected from two main locations: the Vava’u Group of islands, Tonga, and from eastern Australia (Table 1). All necessary permits were obtained for the described field studies and studies did not involve endangered or protected species. Stranded pumice was also collected and examined from Fiji following strandings that occurred in early October, 2006 [12], [13], but lacked epibionts. Floating pumice raft material was collected and examined from Tongan waters and around Home Reef volcano in February 2007. Stranded pumice deposits from eastern Australia were sampled within 1 m^2^ quadrats over pumice strandlines on beaches – this was to provide a representative and achievable sampling of the stranded pumice material given the volume of pumice deposited and length of coastline (>2500 km) along which stranding occurred. At other locations, samples of stranded pumice were collected but over a larger area of the beach and these are referred to as “representative” in Table 1. Beaches were surveyed from January to April 2007 to monitor any influx and stranding of pumice. Pumice strandings along the eastern Australian coast began in late March in far north Queensland, but the primary stranding event along the Queensland and New South Wales coastline began on April 16, 2007 as a result of a change to easterly and northeasterly onshore wind conditions and king tides. Pumice was then collected from all sites listed in Table 1 between April 29 and May 7, 2007. Pumice was additionally collected from Broadbeach (southeast Queensland) between December 27, 2007 and January 2, 2008 following a secondary stranding event resulting from similarly strong onshore wind conditions at this time. These latter samples have provided constraints on the temporal evolution of the rafts and attached biota. Minor pumice strandings, particularly along the southern Queensland and northern New South Wales coastline continued until mid-2008 (∼2 years after the eruption), attesting to the long transport duration and ability of pumice to remain afloat for years.

### Pumice and Biota Description

The number of clasts was counted for each sample site listed in Table 1 and typical bulk pumice samples averaged ∼970 clasts m^−2^. More than 4900 clasts have been individually examined, measured and described – this includes material preserved in alcohol (N = 505), or dried (N = 4479). Epibionts were divided into two basic groupings: colonial (e.g., macroalgae, cyanobacteria, calcareous algae, cheilostome Bryozoa) for which percent coverage of individual pumice clasts was visually estimated, or solitary, where individuals could be counted per pumice clast (e.g., gastropods, goose barnacles, molluscs, arthropods). For each clast, the following data were collected following examination using a binocular microscope: 1) maximum and minimum clast lengths; 2) pumice textural type; 3) evidence for recent clast breakage; 4) biological keel development and location of attachment/occurrence of organisms to either the dorsal or ventral sides of the pumice; 5) total number of plant and invertebrate species; 6) % epibiont coverage of pumice clast; 7) for cyanobacteria, fleshy/macroalgae, calcareous algae, cheilostome Bryozoa - occurrence, % coverage, number of species, types; 8) for gastropods, goose barnacles, bivalves - occurrence, number of species, number of individuals, types and shell lengths; 9) for corals, acorn barnacles and anemones - occurrence, number of species, number of individuals, diameter; 10) for serpulids - occurrence, number of individuals; 11) for forams, arthropods, nudibranchs, sponges, isopods/amphipods and egg casings - occurrence, number of species, number of individuals, types; and 12) for hydroids/scyphozoans - occurrence, number of species, types, and % coverage.

Virtually all pumice clasts were highly abraded and rounded, and most clast abrasion predated epibiont recruitment as the epibionts have grown over abraded surfaces. Later abrasion affected pumice samples washed across reefs, and strand samples collected from reef atolls (e.g., Lady Musgrave, see Fig. 6) tended to have lower occurrences and reduced coverage by soft-tissued epibionts.

Attached biota have been determined to the best workable identifiable taxonomic units; species identification for several taxa requires soft parts, which were not present or preserved on the pumice clasts (e.g., serpulids, scyphozoa). In other cases, species level identification could not be made due to the very juvenile forms present on clasts and this has been a particular issue for the attached corals – the rapid transit and stranding of the pumice limited the growth time available, and virtually all coral spats observed on pumice collected in April-May 2007 were <2 mm in diameter. In addition, little taxonomic work and species documentation are presently available for many of the attached biota for the SW Pacific and Eastern Australia with which to compare.

### Pumice Clast Abundance Estimation

Numbers of pumice clasts produced by the eruption have been estimated in the following way. Discrete Element [43] simulations of spheres settling under gravity were used to estimate the number of clasts comprising the pumice raft of given volume. A pumice raft volume of 0.16 km^3^ is based on the measured areal extent [13] of 1600 km^2^ and an estimated raft thickness of 10 cm. Spheres were initially inserted at random locations within a prismatic volume surrounded by fixed bounding walls. The distribution of sphere diameters matched the measured maximum linear dimensions of 4,875 clasts obtained from locations given in Table 1. A numerical simulation was conducted to settle the spheres under gravity with viscous damping to ensure the spheres came to rest. The volume of the settled sphere assembly was then measured, yielding a number density of 15,793 spheres m^−3^. Using this number density, a pumice raft of 0.16 km^3^ would contain a minimum of 2.5×10^12^ clasts. Since the diameters of the spheres were given by the maximum linear dimensions of measured clasts, this value is considered a lower bound on the number of clasts comprising the pumice raft.

### Pumice Raft Trajectory

The trajectories of the pumice rafts were calculated as a combination of the surface currents and the direct action of winds and waves on the rafts. The surface currents are derived using the methodology of Bonjean & Lagerloef [44]. In this method the surface currents are a combination of wind-driven (Ekman) currents and currents induced by changes in the sea surface height (SSH) and the Coriolis force (geostrophic), along with a small sea surface temperature correction. Due to limitations of the geostrophic assumption and tidal influences, velocity vectors can only be calculated for deep water where bottom drag is not important on the surface current dynamics. The winds were from the final global data assimilation (FNL) run of the Global Forecast System at the National Centers for Environmental prediction in the USA. The sea surface height anomalies were derived from a number of satellites at the Centre National d’Etudes Spatiales (CNES) in France. More detail on the calculation of the trajectories can be found in Bryan et al. [4].

### Ordination Analysis

We conducted an analysis of similarity (ANOSIM) using a Bray-Curtis similarity metric and 9999 permutations using Primer 6 (Plymouth Routines in Multivariate Ecological Research, Plymouth UK; [45], [46]). ANOSIM allowed us to compare presence and absence of species (richness) and their relative abundances to evaluate how communities were changing depending on the collection/arrival time of the pumice rafts (early, middle, and late).

## Supporting Information

Figure S1 **Animated trajectory model of the 2006–2007 Home Reef pumice rafts, based on the integrated surface velocity field (see also** **Fig. 2** **).** Details on the calculation of the pumice trajectories are given in the Materials and Methods section. Grey areas without bathymetric information represent continental shelves of <1000 m depth, where geostrophic ocean currents were not calculated.
(GIF)

Appendix S1 **Data sources to historical (<200 a) pumice raft-producing eruptions shown in** **Figure 1** **.**
(DOCX)
