# Supplementary figures and images for: Rapid, Long-Distance Dispersal by Pumice Rafting
Source: PLoS One. 2012 Jul 18;7(7):e40583. doi: 10.1371/journal.pone.0040583 (PMC3399893; doi:10.1371/journal.pone.0040583)

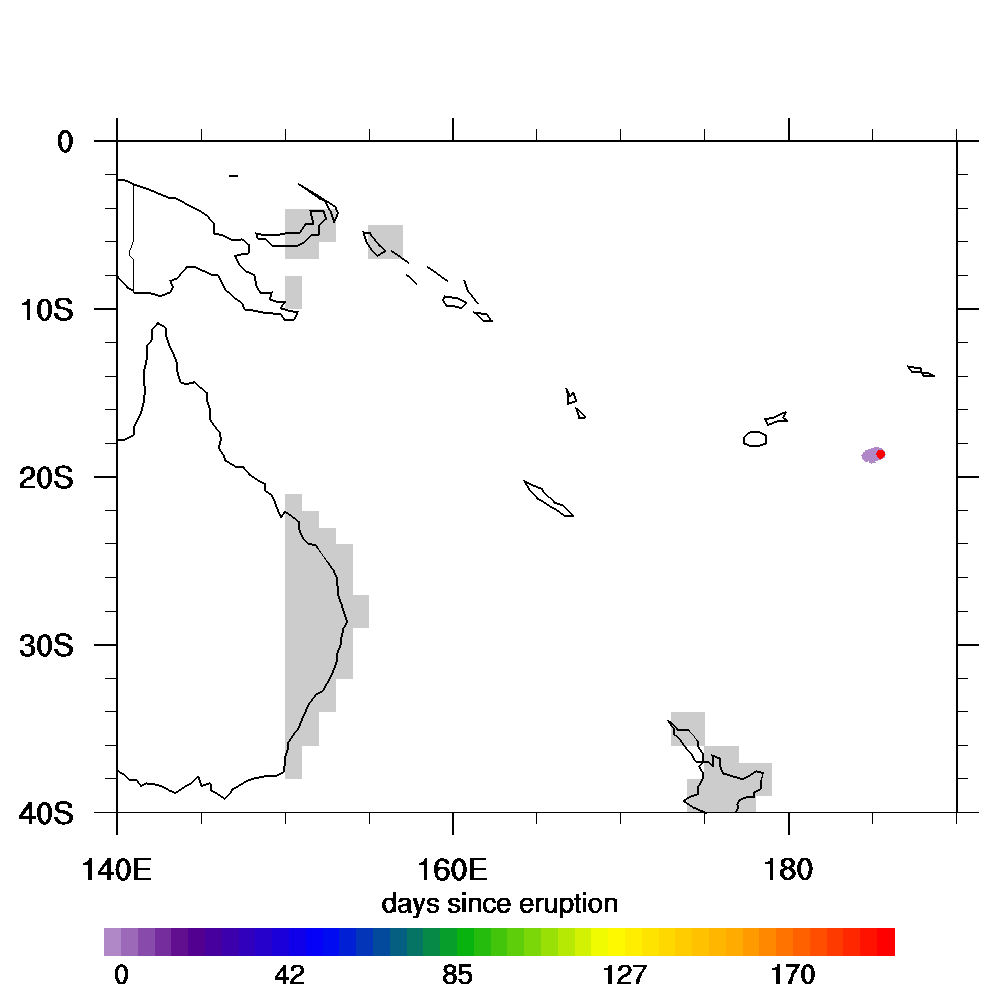

Supplement: Figure S1 — Animated trajectory model of the 2006–2007 Home Reef pumice rafts, based on the integrated surface velocity field (see also Fig. 2 ). Details on the calculation of the pumice trajectories are given in the Materials and Methods section. Grey areas without bathymetric information represent continental shelves of <1000 m depth, where geostrophic ocean currents were not calculated. (GIF) [file pone.0040583.s001.gif]
