# Supplementary material for: Rapid, Long-Distance Dispersal by Pumice Rafting
Source: PLoS One. 2012 Jul 18;7(7):e40583. doi: 10.1371/journal.pone.0040583 (PMC3399893; doi:10.1371/journal.pone.0040583)
Supplement: Appendix S1 — Data sources to historical (<200 a) pumice raft-producing eruptions shown in Figure 1 . (DOCX) [file pone.0040583.s002.docx]

**Supporting Information - Rapid, Long-Distance Dispersal by Pumice Rafting**

**Appendix S1.**

Data sources to historical (<200 a) pumice raft-producing eruptions.

***1. 1815 Tambora eruption, Indonesia***.

Oppenheimer C (2003) Climatic, environmental and human consequences of the largest known historic eruption: Tambora volcano (Indonesia) 1815. Progress in Physical Geography 27: 230. DOI: 10.1191/0309133303pp379ra.

***2. 1883 Krakatau eruption, Indonesia***.

Simkin T, Fiske RS (1983) Krakatau 1883 Eruption and its Effects. Smithsonian Institution Press, Washington, DC, 464 pp.

Jokiel PL, Cox EF (2003) Drift pumice at Christmas Island and Hawaii: evidence of oceanic dispersal patterns. Marine Geology 202: 121-133.

***3. 1952 Volcan Barcena eruption, Isla San Benedicto, Mexico***.

Richards AF (1958) Transpacific distribution of floating pumice from Isla San Benedicto, Mexico. Deep-Sea Research 8: 29-35.

***4. 1952-1953 Myojinsho eruption, Japan***.

Fiske RS, Cashman KV, Shibata A, Watanabe K (1998) Tephra dispersal from Myojinsho, Japan, during its shallow submarine eruption of 1952–1953. Bulletin of Volcanology 59: 262–275.

***5. 1962 South Sandwich Islands eruption (Protector Shoal), British overseas territory*.**

Gass IG, Harris PG, Holgate MW (1963) Pumice eruption in the area of the South Sandwich Islands. Geological Magazine, 100: 321-330.

Sutherland FL (1965) dispersal of pumice, supposedly from the 1962 South Sandwich Islands eruption on southern Australian shores. Nature 207: 1332-1335.

Risso C, Scasso RA, Aparicio A (2002) Presence of large pumice blocks on Tierra del Fuego and South Shetland Islands shorelines, from 1962 South Sandwich Islands eruption. Marine Geology 186: 413– 422.

***6. 1962 Surtsey eruption, Iceland*.**

Gudmundsson F, Ingolfsson A (1967) Goose barnacles (Lepas spp.) on Surtsey pumice. Náttúrufraedingurinn 37: 57-60.

***7. 1964 unknown eruption, Tonga***.

Bryan WB (1868) Low-potash dacite drift pumice from the Coral Sea. Geological Magazine 105: 431-439.

Bryan WB (1971) Coral Sea drift pumice stranded on Eua Island, Tonga, in 1969. Geological Society of America Bulletin 82: 2799-2812.

***8. 1967(-1968) Metis Shoal eruption, Tonga*.**

Bryan WB (1971) Coral Sea drift pumice stranded on Eua Island, Tonga, in 1969. Geological Society of America Bulletin 82: 2799-2812.

Smithsonian Institution (1968) Metis Shoal. Center for Short-Lived Phenomena Report 02-67.

***9. 1973 Curacoa eruption, Tonga*.**

Smithsonian Institution (1973) Curacoa. Center for Short-Lived Phenomena, Event Notification Report 94-73.

***10. 1979 Metis Shoal eruption, Tonga*.**

Smithsonian Institution (1979) Metis Shoal. Scientific Event Alert Network (SEAN) Bulletin 04:05.

**11. 1983 unknown eruption, Kermadec Islands.**

Smithsonian Institution (1983) Floating Pumice (Kermadec Islands). Scientific Event Alert Network (SEAN) Bulletin 08:04-08:08.

***12. 1984 Home Reef eruption, Tonga***.

Smithsonian Institution (1984) Home Reef. Scientific Event Alert Network (SEAN) Bulletin 09:07 – 09:10.

***13. 1986 Fukutoku-okanoba eruption, Japan*.**

Smithsonian Institution (1986) Pumice from unknown source clogs ship's intakes. Scientific Event Alert Network (SEAN) Bulletin 11:12.

***14. 1990 eruption from unknown volcano, Tonga***.

Smithsonian Institution (1990) 30-km zone of pumice from unknown source. Bulletin of the Global Volcanism Network 15:10.

***15. 1995 (-1999) Montserrat eruption, British overseas territory***.

Donovan SK (1999) Pumice and pseudoplankton. Caribbean Journal of Science 35: 323–324.

***16. 2001 Volcano 0403-091 eruption, Tonga***.

Bryan SE, Cook A, Evans J, Colls P, Lawrence M, Wells M, Jell JS, Greig A, Leslie R (2004) Pumice rafting and faunal dispersion during 2001-2002 in the southwest Pacific: record of a dacitic submarine explosive eruption from Tonga. Earth and Planetary Science Letters 227: 135-154.

***17. 2004 Tristan de Cunha eruption, British overseas territory***.

Reagan MK, Turner S, Legg M, Sims KWW, Hards VL (2008) ^238^U- and ^232^Th-decay series constraints on the timescales of crystal fractionation to produce the phonolite erupted in 2004 near Tristan da Cunha, South Atlantic Ocean. Geochimica et Cosmochimica Acta 72: 4367–4378.

***18. 2006 Home Reef eruption, Tonga***.

This study.

Vaughan RG, Abrams MJ, Hook SJ, Pieri DC (2007) Satellite observations of new volcanic island in Tonga. EOS Transactions of the American Geophysical Union 88: 4, 37,41.

***19. 2007 Jebel at Tair eruption, Yemen***.

Smithsonian Institution (2007) Jebel at Tair. Bulletin of the Global Volcanism Network 32:10.

***20. 2008 Chaiten eruption, Chile***.

Smithsonian Institution (2008) Chaiten. Bulletin of the Global Volcanism Network 33:06.
